# Histological changes in the human esophagus following triamcinolone injection to prevent esophageal stricture after endoscopic submucosal dissection

**DOI:** 10.1007/s10388-021-00818-0

**Published:** 2021-03-02

**Authors:** Yudai Kawamura, Kenro Kawada, Takashi Ito, Katsumasa Saito, Naoto Fujiwara, Takuya Okada, Akihiro Hoshino, Yutaka Tokairin, Yasuaki Nakajima, Tatsuyuki Kawano, Masanori Tokunaga, Yusuke Kinugasa

**Affiliations:** 1 Department of Gastrointestinal Surgery, Graduate School of Medical and Dental Sciences (Medicine), Tokyo Medical and Dental University, 1-5-45 Yushima, Bunkyo-ku, Tokyo, 113-8510 Japan; 2 Department of Surgery, Tokyo Metropolitan Health and Hospitals Corporation Toshima Hospital, Tokyo, Japan; 3 Department of Human Pathology, Graduate School of Medical and Dental Sciences (Medicine), Tokyo Medical and Dental University, Tokyo, Japan; 4 Department of Surgery, Edogawa Hospital, Tokyo, Japan; 5 Department of Surgery, Soka Municipal Hospital, Saitama, Japan

**Keywords:** Triamcinolone acetonide, Esophageal cancer, Esophageal stenosis, Esophagectomy

## Abstract

**Background:**

Locoregional steroid injection prevents post-endoscopic submucosal dissection (ESD) esophageal stricture, but histological changes that occur following steroid injection in the human esophagus are unclear. This study investigated the histopathological characteristics caused by locoregional triamcinolone acetonide (TA) injection using human esophagectomy specimens.

**Methods:**

From January 2014 to December 2019, among 297 patients (373 lesions) who underwent esophageal ESD, 13 patients who underwent additional esophagectomy after ESD were examined. Seven patients (TA group) with wide excisions were injected with TA after ESD and another six patients (Non-TA group) with smaller tumors were not injected with TA. The clinical background of these patients and histopathological features of ESD ulcer scar obtained from esophagectomy specimens were retrospectively investigated.

**Results:**

The circumferential rate of ESD excision was more than three-quarters in all cases in the TA group, whereas it was less than three-quarters in the Non-TA group. No other statistical difference in the clinical background was found between the two groups. The subepithelial fibrous tissue of the ESD ulcer scar in the TA group was significantly thinner than that in the Non-TA group (*P* < 0.05). There was no significant difference in the thickness of the regenerated epithelium and muscularis propria layer of the ESD ulcer scar.

**Conclusions:**

Histological finding of thinning of the subepithelial fibrous tissue of ESD ulcer scar in the human esophagus after TA injection was obtained. This suggests that TA suppresses the proliferation of the fibrous tissue of the subepithelial layer to help prevent esophageal stricture after widespread ESD in the human esophagus.

**Supplementary Information:**

The online version contains supplementary material available at 10.1007/s10388-021-00818-0.

## Introduction

Esophageal cancer is the ninth most common malignancy in the world [1]. For the treatment of superficial esophageal cancer, with no risk of lymph node metastasis, endoscopic submucosal dissection (ESD) was developed in Japan and has spread worldwide with the progress of endoscopic diagnostic and treatment techniques [2, 3]. Compared to endoscopic mucosal resection (EMR), ESD makes it possible to resect specimen *en bloc* regardless of tumor size, leading to a more precise pathological diagnosis. The clinical outcomes of esophageal ESD are comparable to surgical treatment. Moreover, its risk of complications is lower than that in highly invasive esophagectomy [4–7]. However, esophageal ESD with a wide excision range is associated with a high occurrence of esophageal strictures [8]. Esophageal stricture after widespread ESD is a serious complication that lowers the patient’s quality of life and requires frequent balloon dilatation [9].

Previous studies showed that local steroid injection into the ESD ulcer prevented post-ESD stricture [10–12]. In our institution, when the circumferential rate of ESD ulcer is more than three-quarters, triamcinolone acetonide (TA) is injected locally into the artificial ulcer layer and good clinical results are obtained.

Although TA local injection after widespread ESD may suppress fibrosis and is clinically effective in preventing esophageal stricture, it is not well understood how TA local injection causes histological changes in the human esophagus. The aim of this study was to retrospectively investigate the histological features, such as suppression of fibrosis caused by local TA injection, using the human esophagectomy specimens obtained from the patients who underwent additional surgery after ESD.

## Patients and methods

### Patients

In total, 297 patients with 373 superficial esophageal cancers were treated with ESD at Tokyo Medical and Dental University Hospital from January 1, 2014 to December 31, 2019. Thirteen of these patients (2.95%) with 14 lesions underwent additional radical esophagectomy based on the subsequent pathological diagnosis of the specimens obtained by ESD. Of these, seven patients with seven lesions had received local injection of TA (Kenacort-A 40 mg/1 mL; Bristol-Myers Squibb Co., Tokyo, Japan) to the superficial layer of the residual submucosal tissue of the ESD ulcer bed to prevent esophageal stricture due to extensive resection (circumferential rate ≥ 3/4) during ESD. The remaining six patients with seven lesions were not injected with TA after ESD because the ulcer was small (circumferential rate < 3/4). The esophagectomy specimens obtained from these 13 patients were histologically examined retrospectively. All of these patients had not received any treatment, such as chemotherapy or radiation therapy, prior to ESD.

### TA injection

**Fig. 1:**
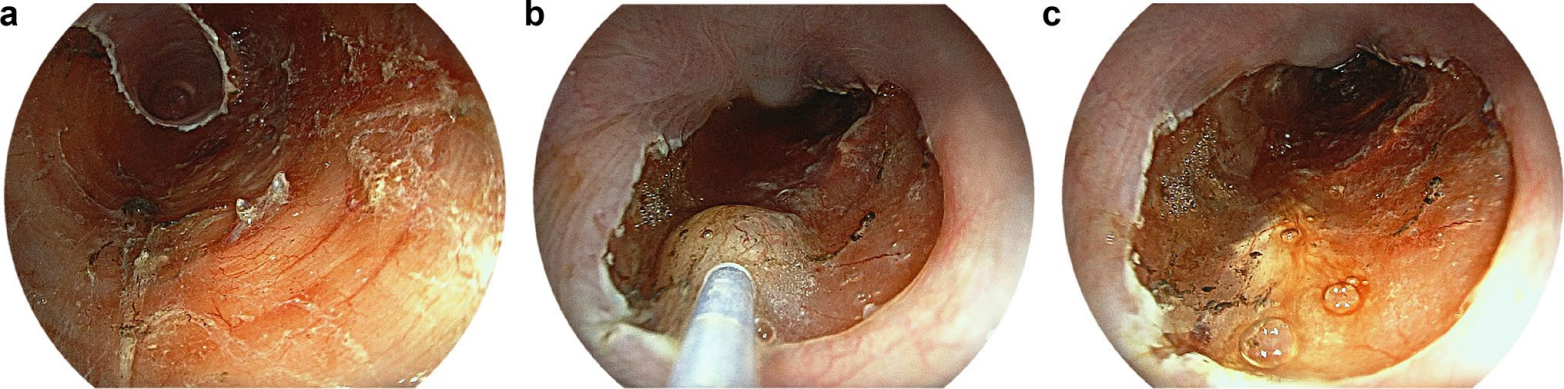
Representative endoscopic appearance of TA injection after ESD. **a** A subcircumferential artificial ulcer was created after ESD. The muscularis propria layer was found under the residual submucosal layer. **b** After the ESD, TA was injected into the submucosal layer of the ulcer site, being careful not to inject deeper than the muscularis propria layer. **c** White suspended liquid was stored in the residual submucosal tissue. *ESD* endoscopic submucosal dissection, *TA* triamcinolone acetonide

Except for one case where TA was injected the day after ESD, a single session of locoregional TA injection was performed immediately after ESD in all cases. The TA injection procedure is shown in Fig. 1. TA (Kenacort-A 40 mg/1 mL) was injected into the superficial layer of the ESD ulcer bed, using a 25-gauge or 26-gauge endoscopic needle at 0.1 mL each time for a total of 1 mL. Since there have been reports of periesophageal abscess formation or delayed esophageal perforation after TA injection [13–15], we were careful not to inject deeper than the muscularis propria layer.

### Clinical and histopathological survey

The clinical background of these 13 patients was retrieved from their medical records and endoscopic reports. We investigated sex, age, tumor location, interval between ESD and operation, stricture rate, macroscopic type of tumor, tumor histology, depth of tumor invasion, lymphovascular invasion, dissection margin, size of ESD specimen, tumor remnant, lymph node metastasis, and size of ESD scar. In addition, the histopathological evaluation was performed retrospectively using the radical esophagectomy specimens, in which the ESD ulcer scars existed, to determine characteristic findings such as suppression of fibrosis in ulcer scars attributable to TA injection. The esophagectomy specimens were cut into 3- to 4-mm slices, fixed with formalin, embedded in paraffin, and then sliced into 3-μm-thick sections for hematoxylin–eosin (HE) and Elastica van Gieson (EVG) staining. These histopathological specimens had been prepared at the Department of Pathology, Tokyo Medical and Dental University Hospital, and were used for pathological diagnosis. Masson trichrome (MT) staining and desmin immunohistochemical staining were performed for more detailed histological observation. The primary antibody to desmin (clone D33) was obtained from MONOSAN (Concord, CA, USA). The antibody was used at a dilution of 1:50. Immunohistochemical analysis was performed using a Leica Bond III automated immunostainer (Leica, Bannockburn, IL, USA). BOND Epitope-Retrieval Solution 2 (AR9640) was used for 20 min before application of the primary antibody (15 min). Detection was done using Leica Bond-detection kit (DS9800).

Histopathologically, ESD ulcer scars were defined as the area of the muscularis mucosal defects in esophagectomy specimens. Regenerated epithelium (ReEp) and fibrous tissue proliferation were observed there. The layer between the ReEp and the muscularis propria (MP) in the ulcer scar is referred to as the subepithelial layer or subepithelial fibrous tissue (SF).

All sections of the ulcer scar were observed under a microscope to extract histological characteristics, focusing on the degree of fibrosis. The thickness of each layer (ReEp, SF and MP) in the esophageal wall at the center of the ulcer scar were measured using the software CellSens (Olympus, Tokyo, Japan). These clinicopathological characteristics of patients who received TA injection after ESD (TA group) were compared with those who did not receive TA injection (Non-TA group).

### Statistical analyses

All statistical analyses were performed with EZR, version 1.42 (Saitama Medical Center, Jichi Medical University, Saitama, Japan), which is a graphical user interface for R (The R Foundation for Statistical Computing, Vienna, Austria). More precisely, it is a modified version of R commander designed to add statistical functions frequently used in biostatistics [16]. Categorical data were analyzed with Fischer’s exact test, while continuous data were analyzed with Mann–Whitney *U* test. The differences with *P* < 0.05 (two-sided) were considered statistically significant.

## Results

### Baseline characteristics of the patients and tumors

Table 1 shows the baseline characteristics of the patients and tumors analyzed. The study included 14 lesions of 13 patients (12 men and 1 woman) who underwent additional esophagectomy after ESD without prior treatment. The patients were aged from 54 to 78 years (median 65 years). Twelve cases had squamous cell carcinoma (SCC), and there was one case each of basaloid squamous cell carcinoma (BSCC) and carcinosarcoma. Additional esophagectomies in the TA group were performed because of pT1a-MM with ly1 (one case), pT1a-MM with VM1 (one case), pT1b-SM1 with a positive stump (one case), and pT1b-SM2 (four cases). In the Non-TA group, one case had pT1a-MM with ly1/v1, one case had multiple lesions (pT1a-LPM and pT1b-SM2), and the remaining cases had pT1b-SM2, so they underwent additional esophagectomy. No statistically significant differences were found in sex, age, tumor location, the interval between ESD and operation, stricture rate, macroscopic type of tumor, tumor histology, depth of tumor invasion, lymphovascular invasion, dissection margin, tumor remnant, and lymph node metastasis between the two groups. In all cases in the TA group, the circumferential rate of ESD excision was more than three-quarters, whereas it was less than three-quarters in all cases in the Non-TA group. The maximum length of the ESD specimen was longer in the TA group (median 60 mm, range 35–75 mm) than in the Non-TA group (median 34 mm, range 17–48 mm, *P* = 0.007). The size of the post-ESD ulcers was significantly larger in the TA group (median 55 mm, range 23–72 mm) than in the Non-TA group (median 25 mm, range 10–40 mm, *P* = 0.015). Detailed clinicopathological findings of all cases are given in supplementary Table 1.

**Table 1:** Baseline clinicopathological characteristics of the patients and tumors analyzed

	TA group	Non-TA group	*P* value
	7 cases, 7 lesions	6 cases, 7 lesions	
Sex, *n* (%)			*0.462*
Male	7 (100)	5 (83.3)	
Female	0 (0)	1 (16.7)	
Age, years, median (range)	65 (64–78)	63 (54–72)	*0.059*
Tumor location, *n* (%)			*0.592*
Ce	1 (14.3)	0 (0)	
UtMt	1 (14.3)	0 (0)	
Mt	2 (28.6)	5 (71.4)	
MtLt	1 (14.3)	0 (0)	
LtMtAe	1 (14.3)	0 (0)	
Lt	1 (14.3)	1 (14.3)	
Ae	0 (0)	1 (14.3)	
Number of days from ESD to operation, Median (range)	85 (63–131)	98 (71–190)	*0.391*
Stricture after ESD, *n* (%)	1 (14.3)	0 (0)	*1*
ESD specimen			
Macroscopic type of tumor, *n* (%)			*1*
Elevated	4 (57.1)	3 (42.9)	
Flat/depressed	3 (42.9)	4 (57.1)	
Histology, *n* (%)			*0.462*
SCC	5 (71.4)	7 (100)	
Others	2 (28.6)	0 (0)	
Depth of tumor invasion, *n* (%)			*1*
pT1a	2 (28.6)	2 (28.6)	
pT1b	5 (71.4)	5 (71.4)	
Lymphovascular invasion positive, *n* (%)	5 (71.4)	6 (85.7)	*1*
Dissection margin positive, *n* (%)	5 (71.4)	2 (28.6)	*0.286*
Size of specimen, mm, median (range)	60 (35–75)	34 (17–48)	*0.007*
Surgical specimen			
Remnant of tumor, *n* (%)	2 (28.6)	1 (14.3)	*1*
Lymph node metastasis positive, *n* (%)	2 (28.6)	2 (33.3)	*1*
Size of scar, mm, median (range)	55 (23–72)	*25 (10–40)*	*0.015*

Italic values indicate significance of *p* value (*P* < 0.05)

*Ce* cervical esophagus, *Ut* upper thoracic esophagus, *Mt* middle thoracic esophagus, *Lt* lower thoracic esophagus, *Ae* abdominal esophagus, *SCC* squamous cell carcinoma, *ESD* endoscopic submucosal dissection, *TA* triamcinolone acetonide

### Clinical course

One case in the TA group required balloon dilatation due to post-ESD esophageal strictures before additional esophagectomy. On the contrary, no post-ESD stricture occurred in the Non-TA group. No other complications due to ESD or local TA injection such as intraoperative or delayed perforation were reported.

### Histopathological findings

**Fig. 2:**
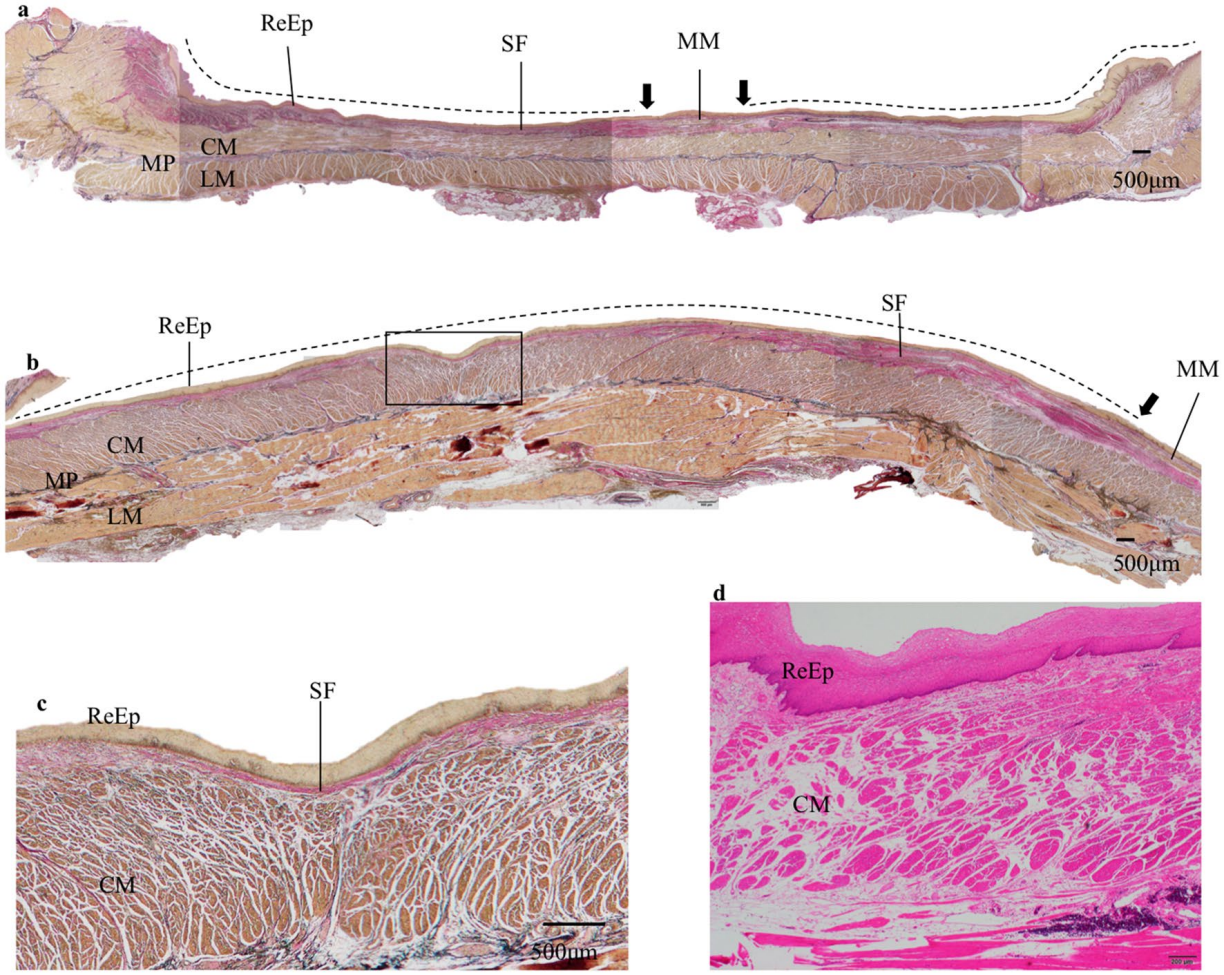
Representative histological findings of the artificial ulcer in esophagectomy specimen after ESD in the TA group. **a** Histological findings of the esophagectomy specimen from one representative case. The black arrow indicates the border of the ESD scar because of the muscularis mucosae disruption. The dotted line indicates the ESD ulcer scar. The subepithelial fibrous tissue is relatively thin over the whole area and the regenerated epithelium and the muscularis propria are close to each other. EVG, original magnification × 20. **b** Histological findings of the central part of an artificial ulcer in an esophagectomy specimen from another case. The black arrow indicates the disruption of muscularis mucosae, the dotted line indicates the ESD ulcer scar. The regenerated epithelium and the muscularis propria layer are very close, and subepithelial fibrous tissue between them is barely observed. EVG, original magnification × 20. **c** Enlarged view of the area surrounded by black squares in Fig. 1b. The subepithelial fibrous tissue is very thin and the regenerated epithelium and the muscularis propria layer are almost in contact with each other. EVG, original magnification × 40. **d** Histological findings of another case. The regenerated epithelium and the muscularis propria layer are almost in contact with each other. HE, original magnification × 40. *ReEp* regenerated epithelium, *MM* muscularis mucosae, *MP* muscularis propria, *CM* circular muscle, *LM* longitudinal muscle, *SF* subepithelial fibrous tissue, *EVG* Elastica van Gieson staining, *HE* hematoxylin and eosin staining, *TA* triamcinolone acetonide

Histopathological findings of the esophagectomy specimens were obtained through retrospective microscopic observation primarily using HE-stained and EVG-stained specimens and supplemented with MT-stained and desmin-stained histopathological examination. The border of the ESD ulcer scar was defined as the disrupted part of the MM. Representative findings of the TA group cases are shown in Fig. 2.

The ulcer scar was covered with the ReEp. In the TA group, overall, the SF of the ulcer scar area was thinned. The proliferation of fibrous tissue in the subepithelial layer was observed in all cases. Of the seven TA group cases, five cases demonstrated a very thin subepithelial layer over a wide area. There was an area where the ReEp was in contact with the MP layer. This characteristic finding was found only in cases in the TA group and not in the Non-TA group (Table 2).

**Table 2:** Thickness of subepithelial fibrous tissue

	TA group	Non-TA group	*P* value
	7 cases, 7 lesions	6 cases, 7 lesions	
Lack of or thinning of subepithelial fibrous tissue, *n* (%)			*0.021*
Positive	5 (71.4)	0 (0)	
Negative	2 (28.6)	7 (100)	

Italic value indicate significance of *p* value (*P* < 0.05)

The MP layers were generally maintained, and the circular and longitudinal muscles were clearly observed. Except for one case, changes in the edematous of the MP layer were observed. No other notable findings in the MP layer such as inflammatory cell infiltration and fibrosis were observed.

**Fig. 3:**
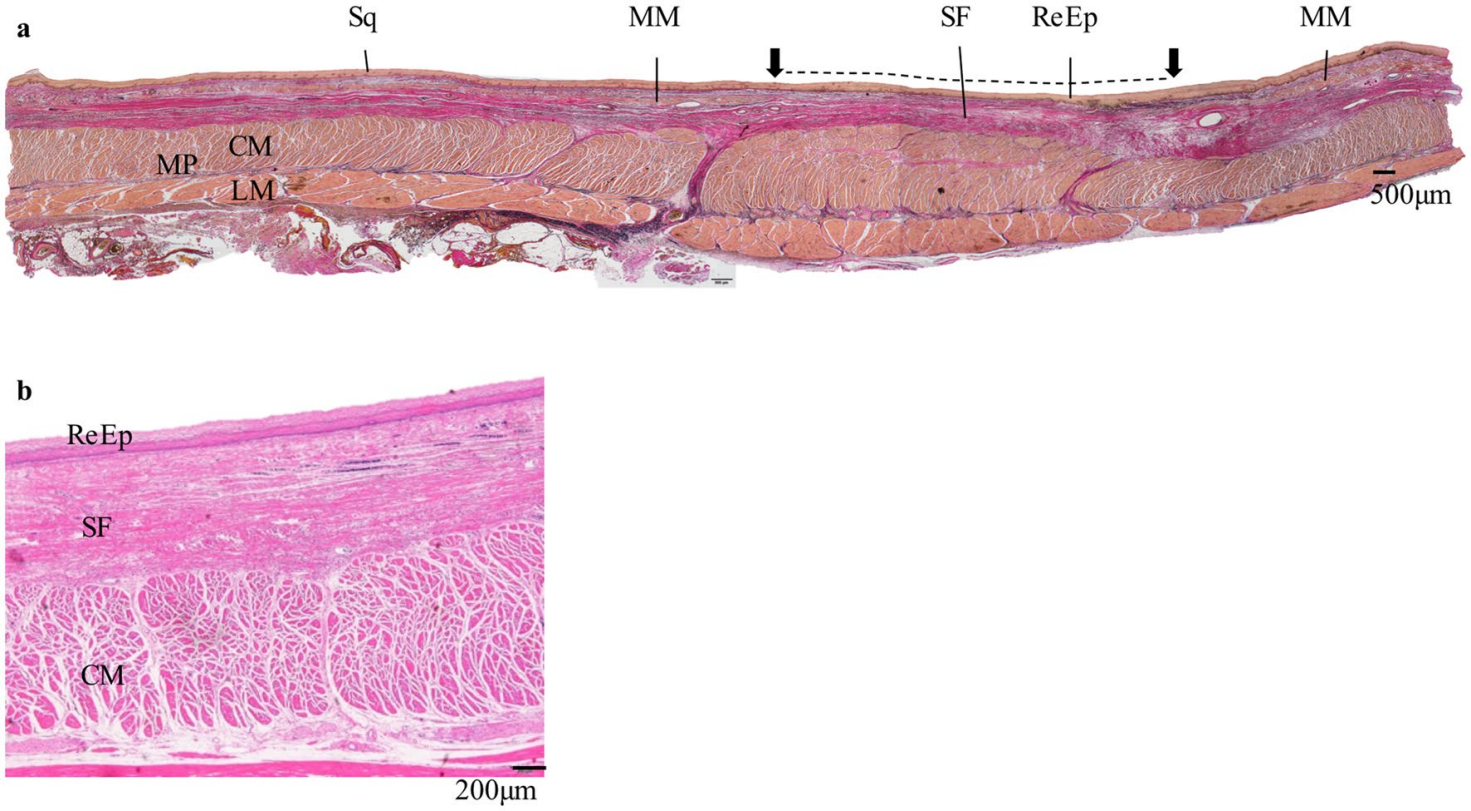
Histological findings of the artificial ulcer after ESD in esophagectomy specimen of the Non-TA group. **a** Histological findings of the resected specimen around the artificial ulcer of one representative case. The disrupted part of the muscularis mucosae indicates the boundary of the artificial ulcer (black arrow). The dotted line indicates the ESD ulcer scar. In the artificial ulcers’ scar, sufficient volume of fibrous tissue is found beneath the regenerated epithelium. The thickness of the muscularis propria layer is not very different from that of the normal esophageal wall. All other Non-TA group specimens had similar subepithelial fibrous tissue thickness. EVG, original magnification × 20. **b** Histological findings of the artificial ulcer of another case in higher magnification. Muscularis mucosae is not found in the area. Under the regenerated epithelium, thick subepithelial fibrous tissue is observed. The muscularis propria layer is thick. HE, original magnification × 40. *Sq* squamous epithelium, *ReEp* regenerated epithelium, *MM* muscularis mucosae, *MP* muscularis propria, *CM* circular muscle, *LM* longitudinal muscle, *SF* subepithelial fibrous tissue, *EVG* Elastica van Gieson staining, *HE* hematoxylin and eosin staining

Figure 3 shows the representative histological findings of the Non-TA group. In the ulcer scar, where the MM was disrupted, thick fibrous tissue was observed throughout the layer beneath the ReEp. In all six specimens with seven ESD ulcer scars, The SF layer was relatively thick; therefore, the ReEp and the MP layer were not close to each other. In the MP layer, characteristic findings were not obtained in terms of its thickness, degree of inflammatory cell infiltration, and fibrosis.

**Fig. 4:**
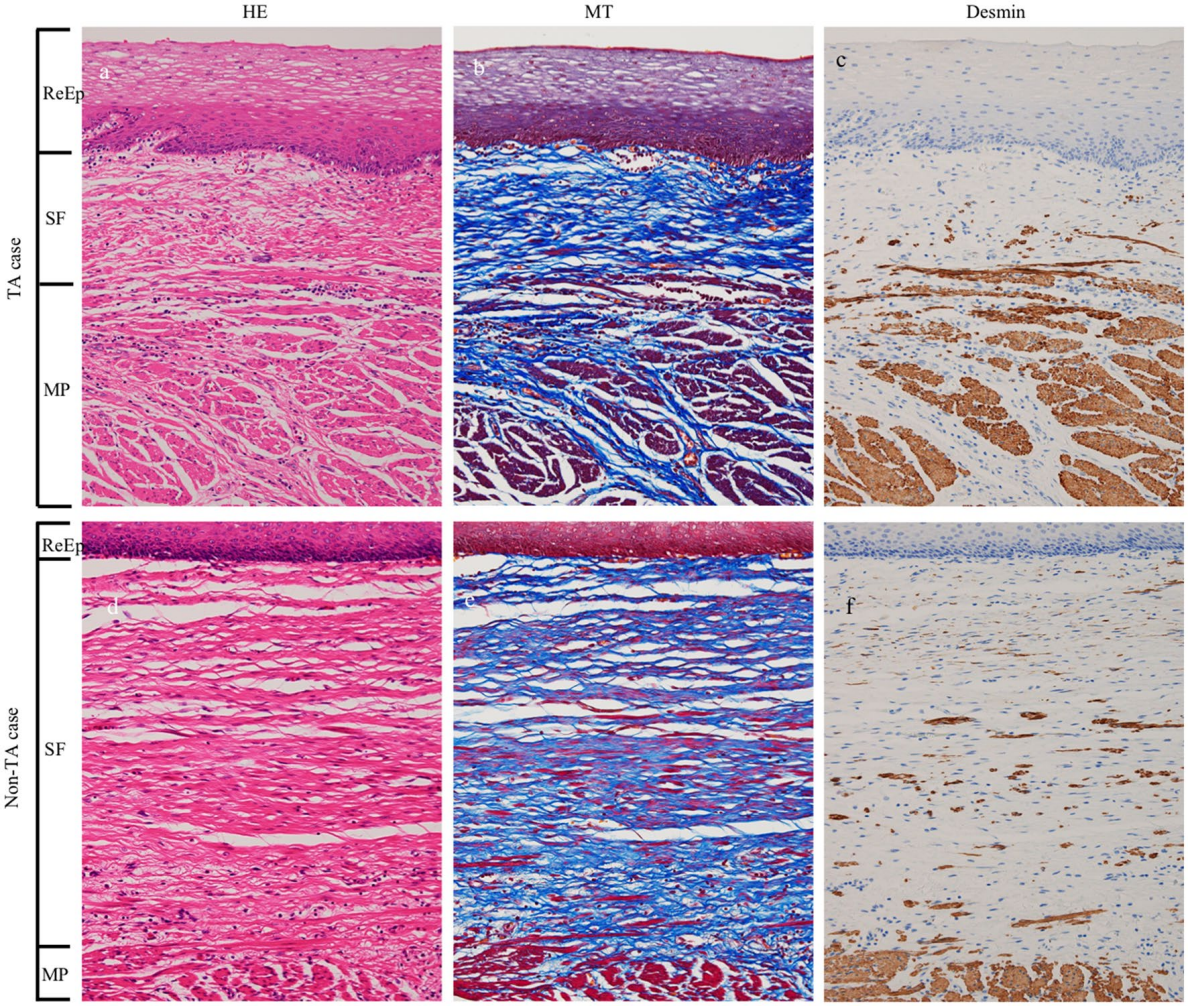
Comparison of histology of the ESD scar in esophagectomy specimens of the representative cases in both TA and Non-TA group. The disappearance of muscularis mucosae and proliferation of fibrous tissue was observed between regenerated epithelium and muscularis propria. In the subepithelial fibrous tissue, the cellularity of fibroblasts/myofibroblasts is low (**a**, **d**) and dense collagen bundles were observed (**b**, **e**). Immunostaining for desmin revealed the mixture of various amounts of smooth muscle fibers between the fibrous tissue (**c**, **f**). The thickness of the subepithelial fibrous tissue in the TA group was less than that in the Non-TA group. *ReEp* regenerated epithelium, *SF* subepithelial fibrous tissue, *MP* muscularis propria, *HE* hematoxylin and eosin staining, *MT* Masson trichrome staining, *TA* triamcinolone acetonide

Figure 4 shows the histology of the ESD scar in esophagectomy specimens of the representative cases in both TA and Non-TA groups. The histology is depicted by HE, MT, and desmin staining.

The disappearance of the MM and proliferation of fibrous tissue was observed between the ReEp and MP layers. In the SF, the cellularity of fibroblasts/myofibroblasts was low (a, d) and dense collagen bundles were observed (b, e). Immunostaining for desmin revealed the mixture of various amounts of smooth muscle fibers between the fibrous tissue (c, f). Thickness of the SF of the TA group was less than that of the Non-TA group.

Histological evaluation of the subepithelial layer is shown in Table 3. The density of collagen fibers and smooth muscle fibers was evaluated in three stages. The number of spindle-shaped cells per high power field (HPF) was also evaluated. Although there were variations among the cases, no statistically significant difference was found between the two groups. The thickness of each layer (ReEp, SF, and MP layer) in the esophageal wall at center of the ulcer scar was measured. The median thickness of the SF layer was 185 μm (range 15–1151 μm) in the TA group, while it was 860 μm (range 753–887 μm) in the Non-TA group. SF layer in the TA group was significantly thinner (*P* = 0.030) than the Non-TA group. No significant difference was observed in the thickness of the ReEp and MP layers between the two groups.

**Table 3:** Histological evaluation

No.	Histological findings of subepithelial fibrous tissue			Thickness of each layer at the center of the ulcer, μm		
	Density of collagen fiber^a^	Density of smooth muscle fiber^b^	Number of spindle stromal cells, /HPF	Regenerated epithelium	Subepithelial fibrous tissue	Muscularis propria
TA group						
1	2	1	189	85	185	2672
2	1	1	126	162	15	2817
3	3	1	270	224	1151	1689
4	2	3	369	209	634	5005
5	2	1	252	132	47	3986
6	2	2	147	231	239	2847
7	2	1	363	67	81	1582
Median (range)	–	–	252 (126–369)	162 (66–231)	185 (15–1151)	2817 (1582–5005)
Non-TA group						
1	2	1	177	81	860	1937
2	2	3	462	410	871	1959
3	2	1	144	144	886	2764
4	1	1	63	112	779	1325
5	1	1	102	114	810	1487
6	3	1	339	261	753	2701
7	2	2	243	163	887	1902
Median (range)	–	–	177 (63–462)	144 (82–410)	860 (753–887)	1937 (1325–2764)
*P* value	*1*	*1*	*0.443*	*0.898*	*0.030*	*0.125*

Italic values indicate significance of *p* value (*P* < 0.05)

^a^Density of collagen fiber: 1, Sparse proliferation of collagen fibers with edema; 2, Dense proliferation of collagen fiber with mild edema; 3, Dense proliferation of collagen fiber without edema

^b^Density of smooth muscle cells: 1, None or small amount of smooth muscle fibers mixed with collagen fibers; 2, Moderate mixture of smooth muscle fibers but less than collagen fibers; 3, Almost the same amount of smooth muscle fibers as collagen fibers

*No.* case number, *HPF* high power field, *TA* triamcinolone acetonide

## Discussion

In the present study, we investigated the histopathological characteristics caused by locoregional TA injection by using the esophagectomy specimens of patients who had undergone additional surgery after ESD. The SF of the ulcer scar in cases with TA injection was thinner than that in cases without TA injection. In five of seven TA cases, the SF of the ESD ulcer scar was almost deficient, and the MP layer and the ReEp were in contact. These findings were not observed in any of the cases without TA injection. No characteristic findings were obtained in terms of the thickness of the ReEp, degree of inflammatory cell infiltration, and thickness of the MP layer. No significant correlation was found between the size of the ESD specimen and that of the ESD ulcer scar in the esophagectomy specimens, regardless of TA injection. These histological findings suggest that locoregional TA injection suppresses the proliferation of the SF and may contribute to the prevention of esophageal strictures after widespread ESD.

Previous studies have reported methods, such as preventive balloon dilatation, steroid administration, esophageal stent treatment, and tissue engineering, to prevent post-ESD esophageal stricture [11, 17–22]. Among these methods, steroid administration to prevent stricture included oral steroid administration, TA injection, and TA-filling method. Since Yamaguchi et al.’s first report [23], oral steroid therapy may have been considered the simplest method, but complications due to long-term use of steroids cannot be ignored. A recent study reported the usefulness of the TA-filling method [24]. In particular, locoregional TA injection therapy is widely accepted for its effectiveness and usefulness as a post-ESD stricture prevention method [10, 22, 25].

Despite TA injection, some patients still develop esophageal strictures after ESD [26, 27]. Okamoto et al. [26] reviewed 53 ESD cases (57 lesions) who received local TA injection to prevent esophageal stricture due to widespread exfoliation of more than two-thirds of the circumference. They reported that the rate of esophageal strictures was highest if the mucosal defect was more than seven-eighths of the circumference; thus, in such cases, alternative strategies, such as application of polyglycolic acid sheets [20], are required. In our study, one patient with TA injection had post-ESD stricture and required balloon dilatation. In this case, the proliferation of the SF was strongly observed (data not shown). The observation that the exfoliation area was particularly large in this case is consistent with the findings of Okamoto et al. [26].

In the fields of dermatology and plastic surgery, it has been established that Kenacort injection is effective in treating keloids [28, 29]. Several basic histopathological studies have revealed that steroid suppresses fibroblast proliferation by controlling chemokines and cytokines [30, 31]. In contrast, to the best of our knowledge, no histological study focused on stricture-preventing effect of steroid injection in human esophagus has been conducted.

Honda et al. [32] performed esophageal EMR of six canine models, observed the ulcer scar histologically immediately after EMR and on postoperative days (POD) 2, 4, 7, 14, and 28, and examined the time course of histological changes during the healing process. They reported that after the inflammatory reaction in the submucosal layer caused by food, digestive juices, saliva, and reflux of gastric acid in addition to EMR itself, epithelial cells proliferated and migrated from the border on POD 7. At 1 month after EMR, the ulcer scar was covered with regenerated squamous epithelium, and myofiber atrophy and fibrosis in the muscularis propria remained.

Nonaka et al. [33] injected TA after esophageal ESD in a pig model and observed esophagectomy specimen immunohistologically over time. They reported that during the healing process of ESD ulcer, the proliferation of myofibroblasts, which are dedifferentiated from myocytes of the muscularis propria and arranged in a parallel fashion in the submucosal layer, may play an important role in esophageal stricture formation. They concluded that TA injection may modify these processes to prevent esophageal strictures.

In our study, characteristic findings such as fibrosis, thickness, and inflammatory cell infiltration in the MP were not obtained, but the thinning of the SF was observed in the TA injection group. These characteristic changes that occurred in the subepithelial layer may be consistent with those observed in the aforementioned basic studies using animal models. These two animal studies [32, 33] suggested that the fibrosis in the submucosal layer of the ESD ulcer scar is related to stricture formation.

The muscularis propria of the pig esophagus is a striated muscle, which may not necessarily be similar to the smooth muscle of the human esophagus, but it may be suggestive of its role in esophageal stricture formation. Further, studies have been conducted in the human gastrointestinal tract for histopathological examination of stenosis formation in Crohn's disease [34]. Suekane et al. reported that accumulation of fibrous tissue was observed in the submucosal layer in all intestinal stenotic lesions in Crohn's disease. They concluded that the proliferation and migration of moderately differentiated intestinal smooth muscle cells from the muscular layers are the underlying pathological mechanisms in stricture formation.

Assuming that the above mechanism is instrumental in the human esophagus, esophageal stenosis may be associated with an increase in myofibroblast dedifferentiation from the muscular layer and proliferation of fibrous tissue in the scar area. It is highly probable that local steroid injection is involved in the suppression of these processes, which may be attributed for the thinning of The SF.

In Nonaka et al.'s study, the longest period from ESD to esophagectomy was 8 weeks. In contrast, in our study, the interval between ESD and surgery was 103.9 ± 35.0 days, which was longer than the observation period of Nonaka et al.’s report. Therefore, the number of immature myofibroblasts might have decreased or disappeared when observing surgical specimens under a microscope. MT staining showed that there was no difference in the number of spindle stromal cells, and myofibroblasts between the two groups. Additionally, owing to the long duration between ESD and esophagectomy, it was difficult to observe the state of myofibroblasts.

Since desmin-positive cells are mixed in the fibrotic tissue of the scar, myofibroblast migration and proliferation of fibrous tissue due to dedifferentiation from the MP might have occurred in the human esophagus. Although this may be consistent with the findings of Nonaka et al. [33] and Suekane et al. [34], further investigation is needed to obtain conclusive results.

This study has some limitations. First, this is a retrospective study conducted in a single institute using past pathological specimens. However, in studies using human specimens, ESD cannot be performed on the premise of additional esophagectomy; thus, examining histological changes over time prospectively appears impossible. Second, the number of cases examined was small because cases of esophagectomy performed as an additional treatment after ESD are few. In our institution, only 4.4% (13 of 297 cases) of the patients underwent additional esophagectomy after ESD during the current observation period. This is a small-scale retrospective study comparing seven patients in the TA group with six in the Non-TA group, and this is preliminary study. Third, the site where TA was actually injected could not be identified histologically despite careful microscopic observations by a skilled pathologist. Roques et al. [35] reported that the injected TA remains locally active for 3–4 weeks. In the present study, the interval from TA injection immediately after ESD to additional surgery was much longer (103.9 ± 35.0 days) than 3–4 weeks, so it was difficult to detect TA injection site histologically.

In conclusion, our study shows a characteristic histopathological finding of thinning of The SF in cases with local TA injection after esophageal ESD. To the best of our knowledge, this is the first histological study using human esophagectomy specimens about TA injection after esophageal ESD. Further investigation such as immunohistological study may help elucidate the basic mechanism on how steroid prevents esophageal stricture after ESD.

## Supplementary Information

Below is the link to the electronic supplementary material.

Supplementary file1 (DOCX 30 KB)
