# Supplementary material for: Histological changes in the human esophagus following triamcinolone injection to prevent esophageal stricture after endoscopic submucosal dissection
Source: Esophagus. 2021 Mar 2;18(3):594–603. doi: 10.1007/s10388-021-00818-0 (PMC8172396; doi:10.1007/s10388-021-00818-0)
Supplement: Supplementary file 1 — Supplementary file1 (DOCX 30 KB) [file 10388_2021_818_MOESM1_ESM.docx]

**Histological changes in the human esophagus following triamcinolone injection to prevent esophageal stricture after endoscopic submucosal dissection**

**Yudai Kawamura^1,2^, Kenro Kawada^1^, Takashi Ito^3^, Katsumasa Saito^1^, Naoto Fujiwara^1^, Takuya Okada^1^, Akihiro Hoshino^1^, Yutaka Tokairin^1,2^, Yasuaki Nakajima^4^, Tatsuyuki Kawano^5^, Masanori Tokunaga^1^ , Yusuke Kinugasa^1^**

^1^ Department of Gastrointestinal Surgery, Graduate School of Medical and Dental Sciences (Medicine), Tokyo Medical and Dental University, Tokyo, Japan

^2^ Department of Surgery, Tokyo Metropolitan Health and Hospitals Corporation Toshima Hospital, Tokyo, Japan

^3^ Department of Human Pathology, Graduate School of Medical and Dental Sciences (Medicine), Tokyo Medical and Dental University, Tokyo, Japan.

^4^ Department of Surgery, Edogawa Hospital, Tokyo, Japan

^5^ Department of Surgery, Soka municipal hospital, Saitama, Japan

**Corresponding author:**

Kenro Kawada

Department of Gastrointestinal Surgery

Graduate School of Medical and Dental Sciences (Medicine)

Tokyo Medical and Dental University

1-5-45 Yushima, Bunkyo-ku, Tokyo 113-8510, Japan

Tel.: 03-5803-5254

Fax: 03-3817-4126

Email: [kawada.srg1@tmd.ac.jp](mailto:kawada.srg1@tmd.ac.jp)

**Supplementary Table 1** Clinicopathological findings of all cases

|  |  |  |  |  |  |  |  |  |  |  | Pathological findings of the ESD specimen | | | | |  | Pathological findings of the surgical specimen | | | | |
| --- | --- | --- | --- | --- | --- | --- | --- | --- | --- | --- | --- | --- | --- | --- | --- | --- | --- | --- | --- | --- | --- |
|  | No. | Age (years) | Sex | Tumor location | Circumferential extent | Macroscopic tumor type | Histology | Stricture after ESD | Oral steroid (total dosage) | Interval between ESD and surgery, days | Depth of tumor invasion | Lympho-vascular invasion | Infiltration | Resected margin | Size of specimen, mm |  | Tumor remnant | Lympho-vascular invasion | Resected margin | Lymph node metastasis, n | Size of scar, mm |
| TA group | 1 | 64 | M | Mt | Three-quarters | 0-IIc | SCC | Did not occur | PSL  480 mg | 131 | pT1a-MM | ly1, v0 | n/a | HM0, VM0 | 60×35 |  | - | n/a | Negative | 0/51 | 60×27 |
|  | 2 | 78 | M | Mt | Sub-circumferential | 0-IIc+Is+IIa | BSCC | Did not occur | - | 106 | pT1b-SM2, 1700μm | ly0, v1 | INFb | HM0, VM1 | 50×47 |  | - | ly0, v0 | Negative | 0/41 | 72×27 |
|  | 3 | 65 | M | Ce | Three-quarters | 0-IIc+IIa | SCC | Did not occur | - | 63 | pT1b-SM2, 800 μm | ly1, v1 | INFb | HM0, VM1, RM1 | 35×34 |  | - | ly0, v0 | Negative | 3/60 | 23×21 |
|  | 4 | 72 | M | LtMtAe | Sub-circumferential | 0-IIa | SCC | One month after ESD | PSL  460 mg | 68 | pT1b-SM2, 300 μm | ly0, v0 | INFb | HM1, VM0 | 75×48 |  | + | ly0, v0 | Negative | 0/58 | 38×28 |
|  | 5 | 65 | M | UtMt | Sub-circumferential | 0-IIc+IIa+Is | Carcino- sarcoma | Did not occur |  | 85 | pT1b-SM2, 500μm | ly1, v1 | INFb | HMX, VM0 | 48×25 |  | + | ly0, v0 | Negative | 1/84 | 44×26 |
|  | 6 | 75 | M | Lt | Sub-circumferential | 0-IIc | SCC | Did not occur | PSL  360 mg | 126 | >pT1b-SM1 | ly0, v0 | INFa | HM1,VM1 | 72×56 |  | - | n/a | Negative | 0/68 | 55×30 |
|  | 7 | 65 | M | MtLt | Sub-circumferential | 0-IIc | SCC | Did not occur | - | 71 | pT1a-MM | ly1, v0 | INFb | HMX, VM1 | 66×55 |  | - | n/a | Negative | 0/25 | 60×50 |
|  |  |  |  |  |  |  |  |  |  |  |  |  |  |  |  |  |  |  |  |  |  |
| Non-TA group | 1 | 65 | M | Lt | <3/4 | 0-IIc | SCC | Did not occur | - | 78 | pT1b-SM2, 320μm | ly1, v0 | INFb | HM0, VM0 | 34×30 |  | - | n/a | Negative | 0/74 | 25×20 |
|  | 2 | 72 | M | Mt | <3/4 | 0-IIa | SCC | Did not occur | - | 91 | pT1a-LPM | ly0, v0 | n/a | HM0, VM0 | 17×11 |  | - | n/a | Negative | 1/34 | 17×15 |
|  |  |  |  | Mt | <3/4 | 0-IIb | SCC | Did not occur |  |  | pT1b-SM2, 700μm | ly0, v1 | INFb | HM0, VM0 | 20×17 |  | - | n/a | Negative |  | 25×13 |
|  | 3 | 57 | F | Mt | <3/4 | 0-IIc | SCC | Did not occur | - | 71 | pT1b-SM2, 700μm | ly1, v1 | INFc | HM0, VM0 | 40×26 |  | - | n/a | Negative | 0/71 | 25×5 |
|  | 4 | 54 | M | Mt | <3/4 | 0-IIc | SCC | Did not occur | - | 105 | pT1b-SM2, 1200μm | ly1, v0 | INFc | HM0, VM0 | 33×28 |  | - | n/a | Negative | 3/53 | 18×8 |
|  | 5 | 64 | M | Mt | <3/4 | 0-IIb+IIa | SCC | Did not occur | - | 133 | pT1b-SM2, 500μm | ly1, v0 | INFb | HM1, VM0 | 48×27 |  | - | ly0, v0 | Negative | 0/70 | 40×25 |
|  | 6 | 62 | M | Ae | <3/4 | 0-IIc+IIa | SCC | Did not occur | - | 190 | pT1a-MM | ly1, v1 | INFb-c | HM1, VM0 | 35×20 |  | + | ly0, v0 | Negative | 0/13 | 10×5 |

Non-TA group comprised seven lesions in six patients.

**Abbreviations:** *TA,* triamcinolone acetonide; *ESD,* endoscopic submucosal dissection; *Ce,* cervical esophagus; *Ut,* upper thoracic esophagus; *Mt,* middle thoracic esophagus; *Lt,* lower thoracic esophagus; *Ae,* abdominal esophagus; *SCC,* squamous cell carcinoma; *BSCC,* basaloid squamous cell carcinoma; *PSL,* prednisolone; *MM,* muscularis mucosae; *SM,* submucosal layer; *ly,* lymphatic invasion; *v,* venous invasion; *INF,* infiltrative growth pattern; *HM,* horizontal margin; *VM,* vertical margin; *RM,* radial margin; *n/a,* not available.
